# Divergent Patterns of Bacterial Community Structure and Function in Response to Estuarine Output in the Middle of the Bohai Sea

**DOI:** 10.3389/fmicb.2021.630741

**Published:** 2021-03-08

**Authors:** Caixia Wang, Haikun Zhang, Pengyuan Liu, Yibo Wang, Yanyu Sun, Zenglei Song, Xiaoke Hu

**Affiliations:** ^1^Key Laboratory of Coastal Biology and Bioresource Utilization, Yantai Institute of Coastal Zone Research, Chinese Academy of Sciences, Yantai, China; ^2^Laboratory for Marine Biology and Biotechnology, Qingdao National Laboratory for Marine Science and Technology, Qingdao, China; ^3^Center for Ocean Mega-Science, Chinese Academy of Sciences, Qingdao, China; ^4^University of Chinese Academy of Sciences, Beijing, China

**Keywords:** estuarine output, bacterial community, Bohai Sea, nutrients, high-throughput sequencing

## Abstract

Understanding environment-community relationships under shifting environmental conditions helps uncover mechanisms by which environmental microbial communities manage to improve ecosystem functioning. This study investigated the microbial community and structure near the Yellow Sea River estuary in 12 stations across the middle of the Bohai Sea for over two seasons to elucidate the influence of estuarine output on them. We found that the dominant phyla in all stations were Proteobacteria, Cyanobacteria, Bacteroidetes, Actinobacteria, and Planctomycetes. Alpha-diversity increased near the estuary and bacterial community structure differed with variation of spatiotemporal gradients. Among all the environmental factors surveyed, temperature, salinity, phosphate, silicon, nitrate, and total virioplankton abundance played crucial roles in controlling the bacterial community composition. Some inferred that community functions such as carbohydrate, lipid, amino acid metabolism, xenobiotics biodegradation, membrane transport, and environmental adaptation were much higher in winter; energy and nucleotide metabolism were lower in winter. Our results suggested that estuarine output had a great influence on the Bohai Sea environment and changes in the water environmental conditions caused by estuarine output developed distinctive microbial communities in the middle of the Bohai Sea. The distinctive microbial communities in winter demonstrated that the shifting water environment may stimulate changes in the diversity and then strengthen the predicted functions.

## Introduction

Microorganisms are key components in marine ecosystems which participate in the nutrient cycles, contaminants degradation, and controlling the marine ecosystem diversity (Fuhrman, 2009; Amaral-Zettler et al., 2010). Bacteria dominate the microbial population mainly through the process of photosynthesis and recycling dissolved organic matter and nutrients, and this process accounts for nearly half of the global marine primary productivity (Benner and Herndl, 2011). During this process, the community structure of planktonic bacteria showed a higher phylogenetic and physiological diversity, with significant transformation and adaptability to the surrounding environment (Follows et al., 2007). They can be the first indicators of sensing the environmental changes especially microbial communities that are extremely sensitive to the pollutant discharge (Weathers et al., 2016; Xie et al., 2018). Many factors exert influence on the microbial community and structure. For example, it was reported that perfluoroalkyl acids contamination was greatly associated with the microbial community in the Pearl River Delta (Chen et al., 2019). In addition, some core species such as *Dechloromonas*, *Anaerolineaceae*, *Crenothrix*, *Dehalococcoidales*, *Desulfuromonadales*, and *Xanthomonadales* presented a co-occurrence pattern with PAH pollution in the riverine environment (Yan et al., 2019). It was indicated that some environmental factors like total nitrogen (Yang et al., 2019), heavy metals (Liu et al., 2019; Zhang et al., 2019), temperature (Meng et al., 2016), salinity (Kaur-Kahlon et al., 2016), and phosphate (Wang et al., 2019) were important regulators in controlling microbial community and function according to multiple statistical analyses. The above mentioned studies has focused on the relationships between environmental factors and microbial community within the estuary, yet the microbial diversity and functions affected by the estuarine output in the open sea near the estuary still are not clear. So, it is necessary to study the relationship among bacterial community, microbial ecological functions, and estuarine output in the Bohai Sea.

River discharge has long been considered to be one of the key components in impacting the coastal ecosystem (Harding et al., 2016). The Yellow River is one of the largest sediment-filled rivers in the world which is located in the north of China (Xu et al., 2007). The Yellow River plume flows across inland areas and run into the Laizhou Bay and the middle of the Bohai Sea even reaching the Bohai Strait, which will have a great impact on the Bohai Sea nutrient level, and to some extent, influence the sedimentary environment and thermohaline circulation of the Bohai Sea (Lin et al., 2001). The Yellow River discharge capacity accounts for almost 75% of the total river discharge which has the largest impact on the Bohai Sea (Liu and Yin, 2010). In addition, nutrient variation in the Bohai Sea has been confirmed to be correlated with riverine input (Wang et al., 2019). Meanwhile, some pollutants produced by human activities, such as heavy metals (Sun et al., 2015), pesticide (Li et al., 2015), and polycyclic aromatic hydrocarbons (Li et al., 2016), can also be brought to the estuary and affect the environment of the Bohai Sea, and these pollutants have a considerable influence on the microbial diversity and function in some systems (Wu et al., 2014; Kammoun et al., 2019; Rodríguez et al., 2019). It was also reported that nitrogen and organic matter loadings impacted the microbial process in the marine system (Seitzinger and Kroeze, 1998).

Estuary with characteristics of changeable freshwater-brackish environment has aroused people’s wide concern. Studies about nitrogen speciation and transformation has been conducted under shifting environmental conditions in the Minjiang River, a typical tidal reach in the southeast of China (Xie et al., 2020). In addition, in a coastal freshwater lake (Haringvliet Lake, the Netherlands), it was found that saltwater intrusions posed a serious impact on the nitrogen transformation process. The ability of denitrification to remove bioavailable nitrogen from aquatic ecosystems was largely limited under the influence of salinization (Laverman et al., 2007). Previous studies about the impact of the estuary on the adjacent sea mainly focused on the nitrogen flux (Sun et al., 2013), nutrient transport (Yu et al., 2019; Xiao et al., 2020), distribution and source of organic matter (Liu et al., 2015), spatial distribution of heavy metals (Cheng et al., 2019), pollutant contents (Yuan et al., 2019), and sediment transport relationship (Naito et al., 2019). Even the studies of microbial community in the Yellow River estuary were limited to the main channel from the cradle to the outlet. However, minimal attention had been paid to the bacterial community structure and function in the middle of the Bohai Sea that was affected by the Yellow River estuarine output. In this study, we investigated the spatiotemporal variation of bacterial communities in the middle of the Bohai Sea during summer and winter, respectively. We aimed to (1) document the spatiotemporal patterns and distributions of bacterial community structure around the middle of the Bohai Sea, (2) investigate the potential functional communities, (3) demonstrate the nutrient pattern in the middle of the Bohai Sea that is influenced by the estuarine output, and (4) determine the relationships between environmental factors and bacterial community structure and function in the middle of the Bohai Sea.

## Experimental Procedures

### Study Area and Sampling Procedures

**FIGURE 1:**
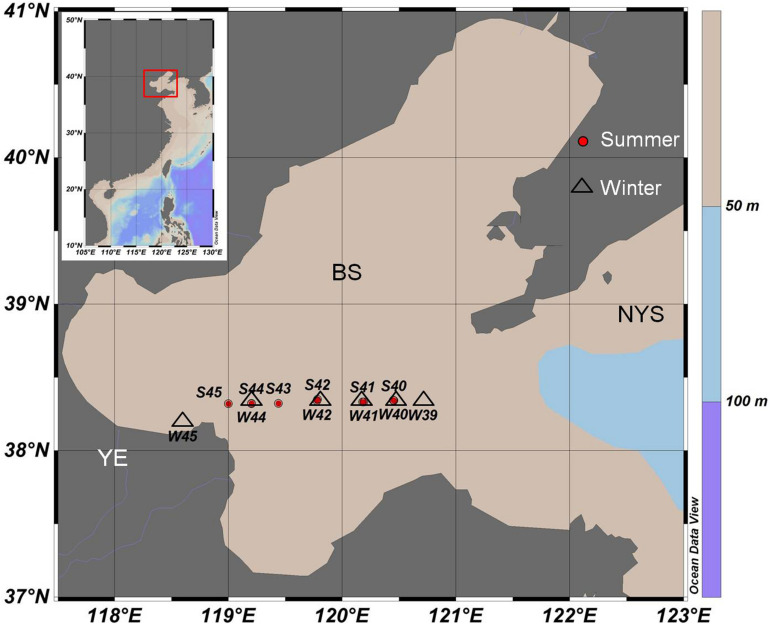
Sampling stations of summer 2015 (red circle, starting with S) and winter 2016 (black triangle, starting with W) in the middle of the Bohai Sea. YE, The Yellow River Estuary; BS, The Bohai Sea; NYS, The North Yellow Sea.

Water samples covered 12 stations (S45, S44, S43, S42, S41, S40, W45, W44, W42, W41, W40, and W39) were collected from August 2015 (summer) to January 2016 (winter) in one transect through the middle of the Bohai Sea (Figure 1). Among all of the stations, site S45 and W45 were the stations that was located near the Yellow River’s mouth. Niskin bottles were used to collect water samples in each station. To evaluate the vertical distribution of virioplankton and bacterioplankton in different depths, a total of 36 water samples were collected from surface (a depth of 3 m below the surface), middle (medium layer with average depth of 13 m), and bottom (a depth of 0.5 m up the benthic zone) levels, respectively. Triplicate samples were taken at each depth. Samples were stored in 2 mL cryovials and then fixed with glutaraldehyde (the final concentration is 5%) immediately. All samples for enumeration of virioplankton and bacterioplankton were first kept at 4°C for 15 min, and then transferred to liquid nitrogen. Samples were transferred to −80°C freezer once back to the laboratory and all samples were analyzed within one month (Wang et al., 2016).

Two liters of surface sea water was filtered through the polycarbonate membranes with 0.22 μm pore size (Millipore, United States) to investigate the bacterial community of the surface water in each station. All these samples were immediately transferred into sterile cryopreservation tube and stored in liquid nitrogen until DNA extraction (Feingersch et al., 2010). Parameters such as water temperature, salinity, and dissolved oxygen (DO) were measured by using the Conductivity Temperature-Depth equipment (CTD) (SBE 25 Sea logger; Sea-Bird Electronic Inc., United States). Approximately 100 mL filtered sea water was collected in the sterile polycarbonate bottles and stored at −20°C for nutrients determination. Concentrations of water nitrate (NO_3_^–^), nitrite (NO_2_^–^), ammonium (NH_4_^+^), phosphate (PO_4_^3–^), and silicate (SiO_3_^2–^) were measured by the nutrient auto-analyzer (AA3; Seal Analytical Ltd., United Kingdom), as previously documented (Tinta et al., 2015).

### Virioplankton and Bacterioplankton Abundance

Triplicate water samples were prepared for enumerating the relative abundance of virioplankton and bacterioplankton by using flow cytometry, as previously described (Paterson et al., 2013). TE buffer (10 mM Tris, 1 mM EDTA) filtered with 0.02 μm membrane was used to dilute water samples. Briefly, samples were stained with SYBR Green I dye (with final concentration of 1:500 stock, molecular probes) first. Virioplankton and bacterioplankton populations were separated and enumerated according to the size and fluorescence based on the forward-angle light scatter (FSC) and side-angle light scatter (SSC) with some modifications (Brussaard, 2004; Seymour et al., 2005). Fully mixed yellow beads with 1 μm particle size were used as reference. All acquired flow cytometer data were processed using FlowJo (TreeStar, San Carlos, CA, United States) in a list-mode folder. The virus to bacteria ratio (VBR) was calculated as the ratio of viral abundance to bacterial abundance.

### DNA Extraction and Illumina HiSeq Sequencing

Bacterial community genomic DNA was extracted by using a PowerSoil DNA Isolation Kit (MoBio, Carlsbad, CA, United States) as the manufacturer described. DNA quantity and concentration were determined by the NanoDrop ND-2000c Spectrophotometer (Thermo Fisher Scientific, Inc., United States). High quality DNA was stored at −80°C for subsequent amplifications. 16S rRNA genes with V4–V5 hypervariable region were amplified by using universal primers 515 F (5′-GTGCCAGCMGCCGCGGTAA-3′) and 909 R (5′-CCCCGYCAATTCMTTTRAGT-3′) with a 12-base barcode sequence to each sample (Tian et al., 2019; Wang et al., 2020). Triplicate PCR products were pooled together with an equal volume in each sample, and 1% agarose gel was run to verify the PCR products. Then, pooled PCR products were sequenced by the TruSeq DNA kit as thee manufacture indicated on Illumina HiSeq 2500 sequencer (Illumina, San Diego, CA, United States). The purified library was constructed according to the requirements of the Illumina library preparation protocol. All processes related to library construction and sequencing were performed by the Novogene Bioinformatics Technology Co., Ltd., Beijing, China. The raw sequencing data related in this study were available at Sequence Read Archive at the website of National Center for Biotechnology Information (NCBI) with BioSample accession No. PRJNA427864.

### Bioinformatics and Data Analysis

According to the barcode sequence and PCR amplification primer sequence, each sample data was separated from the offline data. After cutting the barcode and primer sequence, FLASH was used to assemble the reads of each sample, and the obtained sequence was the original Tags data (Qian et al., 2017). Then, the joined pairs were processed with quality analysis by using QIIME (V1.7.0^1^). In brief, the reads were cut off at any site of more than three successive bases which obtained a Phred quality score (Q) <20. Among them, any reads related ambiguous base calls were deleted (Bokulich et al., 2012). The remaining sequences were filtered to remove chimeras and duplicate sequences with USEARCH (Edgar, 2010). And then, non-chimera unique sequences were clustered into operational taxonomic units (OTUs) at 97% similarity with Uclust algorithm (Edgar, 2010). The classification of OTU taxonomy was performed by the SILVA database 132 (Wang et al., 2007). The sum of each OTU sequence of all samples were calculated. OTUs that contained less than five sequences were screened out and will not be used as a next step analytic target.

Chao1 index, Shannon index, Simpson index, and Ace index were calculated based on the OTU tables by Mothur (version v.1.30.1^2^) to evaluate the alpha diversity of bacteria (Chao et al., 2014). Wilcoxon rank-sum test based on alpha diversity was used to examine the significant differences between different groups. Bar plots of relative abundance of species at different classification level were conducted by R (version 3.1.0). Non-metric multidimensional scaling analysis (NMDS) was conducted to analyze beta diversity of samples. Beta diversity distance matrix was calculated by QIIME. NMDS plot was carried out based on the “vegan” package by R (version 3.1.0) (Datlof et al., 2017). ANOSIM similarity analysis was conducted with the “vegan” package by R (Min et al., 2017). Redundancy analysis (RDA) was performed to visualize the relationship between bacterial community and environmental factors (Urschel et al., 2015). Hierarchical clustering tree on the OTU level was used to identify the differences between different samples according to the distance analysis based on the bray-curtis algorithm (Ward, 1963). Principal co-ordinates analysis (PCoA) was plotted by R based on the selected distance matrix to determine the difference of bacterial community. Spearman correlation analysis was conducted using the SPSS (V.20.0) statistical software. LEfSe (LDA Effect Size) analysis based on non-parametric factorial Kruskal–Wallis (KW) sum-rank test was conducted by LEfSe 1.0 to analyze the biomarkers with statistically significant difference between different groups, i.e., species with significant difference among different groups (Li et al., 2019). The threshold on the logarithmic LDA score for discriminative features was set as 4. The correlation heatmap analysis was mapped by calculating the coefficient between environmental factors and bacterial species to visualize the relationship between environmental factors and bacterial community. Correlation analysis and *P*-value were carried out by using the psych package in R v.3.2.1 (R Core Team, 2015). The correlation coefficients between species and the proportionally normalized OTU tables were imported into the Cytoscape (v3.7.1) software for correlation network construction (Provart, 2012). Species in the top 50 at the genus level and environmental factors (temperature, salinity, DO, phosphate, nitrate, nitrite, ammonium, silicon, total virioplankton, total bacterioplankton, and VBR) were chosen to conduct correlation network. The network topological parameters, including degree of each node, betweenness centrality, closeness centrality, clustering coefficients, and network density, were calculated using the plug-in Network Analyzer in Cytoscape (v3.7.1). Tax4Fun is an open-source R package which was used to predict the function of bacterial 16S amplicon sequencing data (KEGG at the level 1 and level 2) (Aßhauer et al., 2015). Bar chart was carried out by the graph pad 6.0. Contour maps related to the spatial distribution of environmental parameters were conducted by the Surfer 8 based on the longitude and latitude of the sampling sites. The means and deviations of the data were calculated and statistically examined by one-way analysis of variance (ANOVA). Multiple comparison analysis was performed by the SPSS 20.0 (IBM Corporation, United States).

## Results

### Spatiotemporal Variation of Environmental Parameters and Picoplankton Abundance

**FIGURE 2:**
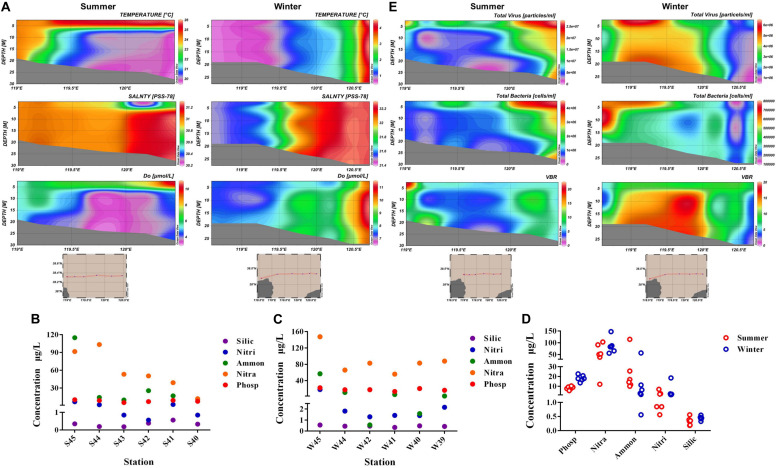
**(A)** Vertical distribution of temperature, salinity, and dissolved oxygen (DO) in summer and winter. **(B)** Concentration of nitrate (NO_3_^–^), nitrite (NO_2_^–^), ammonium (NH_4_^+^), phosphate (PO_4_^3–^), and silicate (SiO_3_^2–^ ) in each station of summer. **(C)** Concentration of nitrate (NO_3_^–^), nitrite (NO_2_^–^), ammonium (NH_4_^+^), phosphate (PO_4_^3–^), and silicate (SiO_3_^2–^ ) in each station of winter. **(D)** Comparison of nutrient concentration between winter and summer. **(E)** Vertical distribution of virioplankton (total abundance of virus), bacterioplankton (total abundance of bacteria), and VBR (virus to bacteria ratio) value in summer and winter (Silic, silicate; Nitri, nitrite; Ammon, ammonium; Nitra, nitrate; Phosp, phosphate).

Environmental parameters were tested in summer and winter of all stations (Supplementary Table 1). Vertical distribution of temperature, salinity, and DO parameters were displayed in Figure 2A. From the horizontal distribution, it was indicated that temperature presented a higher trend in the region far away from the estuary in both summer and winter. While in the vertical distribution, water temperature in the surface layer of summer was much higher than the bottom layer, however, the temperature was similar throughout the water column in winter. Salinity was lower near the estuary than those far away from the estuary in both summer and winter. The average concentration of DO in winter (8.14) was a little higher than the average value in summer (7.21). The concentration of DO exhibited a lower value near the estuary than the areas far away from the estuary (ANOVA, *P* < 0.05). Nutrient concentrations including nitrate (NO_3_^–^), nitrite (NO_2_^–^), ammonium (NH_4_^+^), phosphate (PO_4_^3–^), and silicate (SiO_3_^2–^) present distinct spatial distribution. Concentration of ammonium (NH_4_^+^) and phosphate (PO_4_^3–^) were highest in site S45 and W45 (Figures 2B,C). Concentration of phosphate (PO_4_^3–^) and nitrate (NO_3_^–^) were much higher in winter than in summer (Figure 2D).

Vertical distribution of virioplankton, bacterioplankton, and VBR in winter and summer were showed in Figure 2E. The abundance of virioplankton and bacterioplankton varied greatly in the vertical level during both summer and winter. While in winter, the abundance of picoplankton was much higher near the estuary than the region far away from the estuary (nearly three times) in the horizontal level. The distribution of VBR value was higher in summer near the estuary area, while VBR showed no obvious increase or decrease in trend during winter, only presented its peak value in the middle of the Bohai Sea. In winter, the variation range of virioplankton and bacterioplankton was 7.529 × 10^5^ − 6.449 × 10^6^ particles/mL and 1.261 × 10^5^ − 7.877 × 10^5^ cells/mL. In summer, the variation range of abundance were 7.336 × 10^5^ − 2.703 × 10^7^ particles/mL (virioplankton) and 4.544 × 10^5^ − 4.332 × 10^6^ cells/mL (bacterioplankton), respectively. In winter, the mean abundance of virioplankton and bacterioplankton was 2.863 × 10^6^ particles/mL and 3.481 × 10^5^ cells/mL, respectively. In summer, the average abundance of virioplankton and bacterioplankton was 1.043 × 10^7^ particles/mL and 1.519 × 10^6^ cells/mL, respectively. The abundance of virioplankton and bacterioplankton in summer appeared to be much higher than winter.

### Bacterial Community Composition and Diversity in the Middle of the Bohai Sea

**FIGURE 3:**
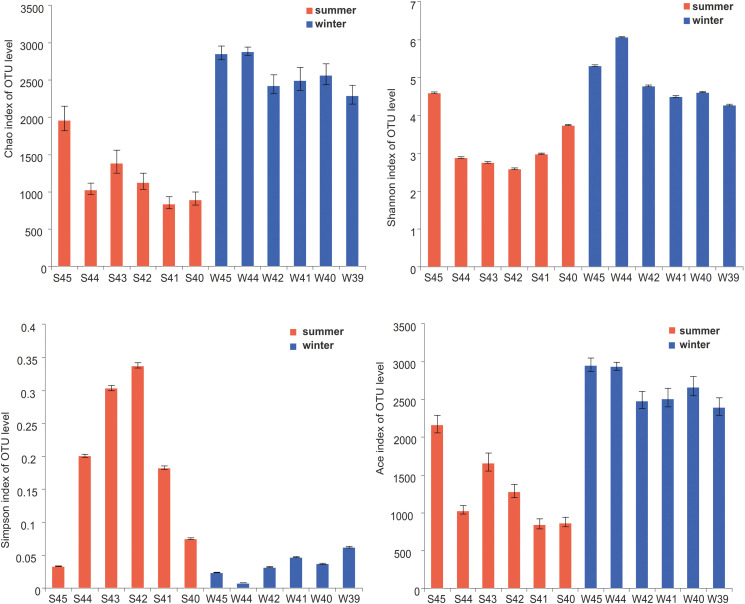
Alpha-diversity indices (Chao1 index, Shannon index, Simpson index, and Ace index) on OTU level were showed at stations in summer and winter. Statistical *t*-test was used to detect whether there were significant differences in index values between two groups. Error bars show the standard deviation from triplicates samples.

**FIGURE 4:**
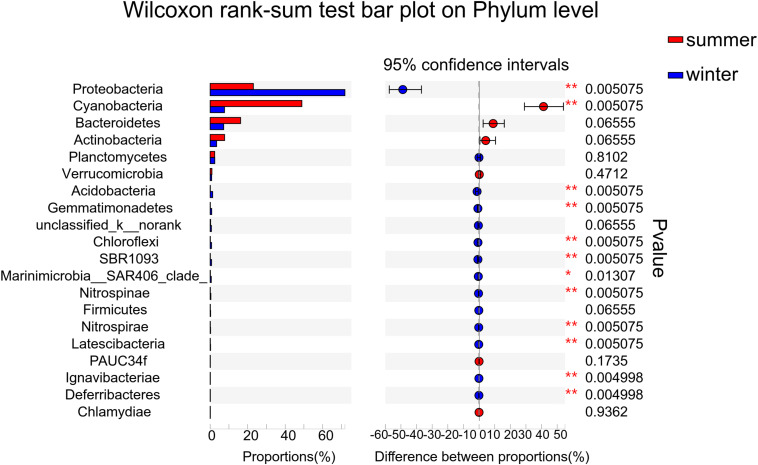
Wilcoxon rank-sum test was used to calculate the differences of key species between summer and winter at the phylum level. The relative abundance of the top 20 phyla was used to determine the differences between the two groups.

According to the alpha diversity results, Chao1 index, Shannon index, and Ace index were found to be much higher in winter than in summer (Figure 3). The distribution trend of the Simpson index was consistent with that of the Shannon index. Bacterial diversity presents a higher trend near the estuary during both summer and winter. The distribution of the Chao1 index and Ace index presented a similar trend in winter and summer. From the spatial distribution, the values of the Chao1 index and Ace index were relatively high near the estuary which indicated that the microbial richness near the estuary was generally higher than that far away from the estuary. Wilcoxon rank-sum test was used to elucidate the index differences between summer and winter (Supplementary Figure 1). It was found that there were significant differences between summer and winter in alpha diversity (*P* < 0.05). Furthermore, the key species were analyzed at the phylum level, which contributed greatly for the differences between summer and winter (Figure 4). Among all of them, Proteobacteria (Wilcoxon rank-sum test, *P* < 0.01), Cyanobacteria (Wilcoxon rank-sum test, *P* < 0.01), Bacteroidetes, Actinobacteria, and Planctomycetes were the phyla that showed significant differences between summer and winter. At the class level (Supplementary Figure 5), Cyanobacteria, Gamaproteobacteria, and Alphaproteobacteria were the main reasons for the differences between summer and winter.

**FIGURE 5:**
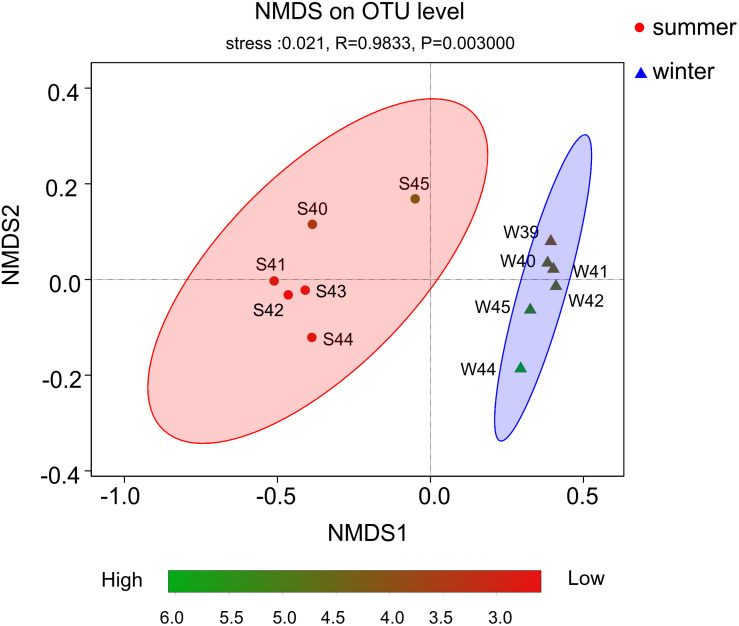
Non-metric multidimensional scaling (NMDS) plot based on OTUs. Color stripe from green to red represent the diversity of Shannon index. The circle and triangle represent seasonal information summer and winter, respectively.

From the result of the NMDS plot (Figure 5), samples were clearly separated into two distinct groups (Supplementary Figures 3, 4). Significant differences between summer and winter were well illustrated by the ANOSIM test (*P* < 0.01, *R* = 0.9833). In summer, bacterial diversity in site S45 was the highest and site S42 was the lowest. While in winter, bacterial diversity in site W44 was the highest and site W39 was the lowest.

**FIGURE 6:**
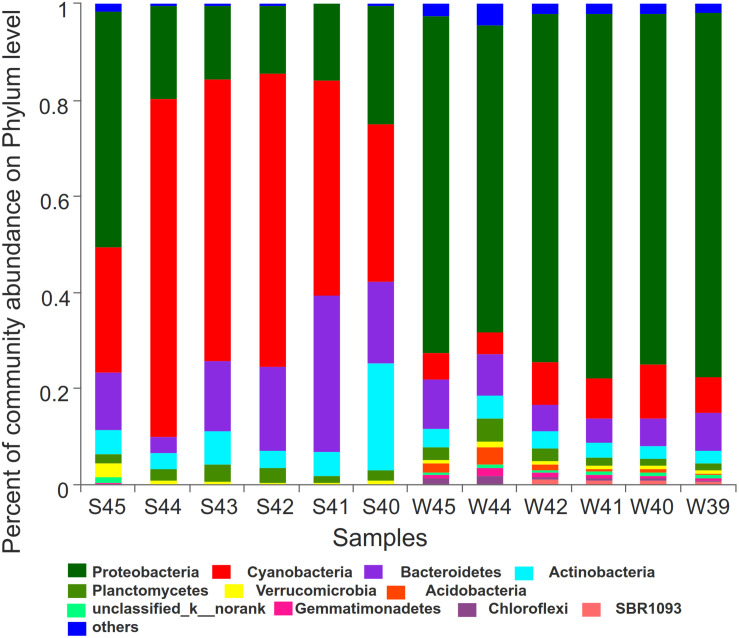
Bar plot was showed to demonstrate bacterial community at the phylum level. The relative abundance of the top12 abundant bacterial classes were used to perform the histogram.

LDA score bar plot was constructed for the taxonomic groups with values lower than 4 to find the unique species in each season (Supplementary Figure 6). The bacterial groups enriched in summer mainly included Subsection I, *Synechococcus*, Cyanobacteria, Sphingobacteriia, Saprospiraceae, and Rhodobacterales. Proteobacteria at the phylum level, Gamaproteobacteria, Betaproteobacteria, and Deltaproteobacteria at the class level, Alteromonadales, SAR11-clade, Oceanospirillales, and Methylophilales at the order level, Pseudoalteromonadaceae at the family level, and *Pseudoalteromonas* at the genus level had the highest LDA scores and represented the leading bacterial members in winter. They were dominant bacterial members responsible for the significant community structural discrepancy following the seasonal shift. Sequences from all 12 station samples were pooled and processed together. A total of 3,544 OTU sequences were generated after clustering at a 97% similarity index and used to analyze the bacterial community structure (Figure 6). Proteobacteria, Cyanobacteria, Bacteroidetes, and Actinobacteria were the dominant bacteria at the phylum level. Cyanobacteria and Proteobacteria were the predominant phyla in summer and winter, respectively. The average related abundance of Cyanobacteria accounted for 48.9% in summer, while the average related abundance of Proteobacteria accounted for 71.9% in winter. Acidobacteria, Gemmatimonadetes, Chloroflexi, and SBR1093 were mainly found in the winter with high relative abundance. From the class level, the main classes of Proteobacteria were Alphaproteobacteria and Gammaproteobacteria in summer. While in winter, the main classes of Proteobacteria were Gammaproteobacteria, Alphaproteobacteria, and Betaproteobacteria (Supplementary Figure 2A). Among all of them, Gammaproteobacteria accounted for the largest proportion of 35.6%. The composition results at the genus level showed that the richness of species was highest at the station S45 near the estuary area in summer (*P* < 0.01). *Synechococcus* with highest abundance of 65.6% in S44 accounted for the largest proportion in summer among all of the stations. In winter, the community composition in stations W45 and W44 was significantly different from those in other stations (*P* < 0.01). The average abundance of *Pseudoalteromonas* was the highest in all stations in winter, and the relative abundance of *Pseudoalteromonas* in W39 station reached 32.1%, accounting for the highest proportion among all stations (Supplementary Figure 2D). Similar to the above, there were significant differences in the composition of bacterial community between winter and summer at the order and family levels (Supplementary Figures 2B,C). At the order level, Alteromonadales was the predominant species in winter group and Subsection I accounted for the largest proportion in summer. At the family level, Pseudoalteromonadaceae was the dominate group in winter and Family I O-Subsection I accounted for the largest proportion in all species.

## Effects of Environmental Factors on Bacterial Community Structure

**FIGURE 7:**
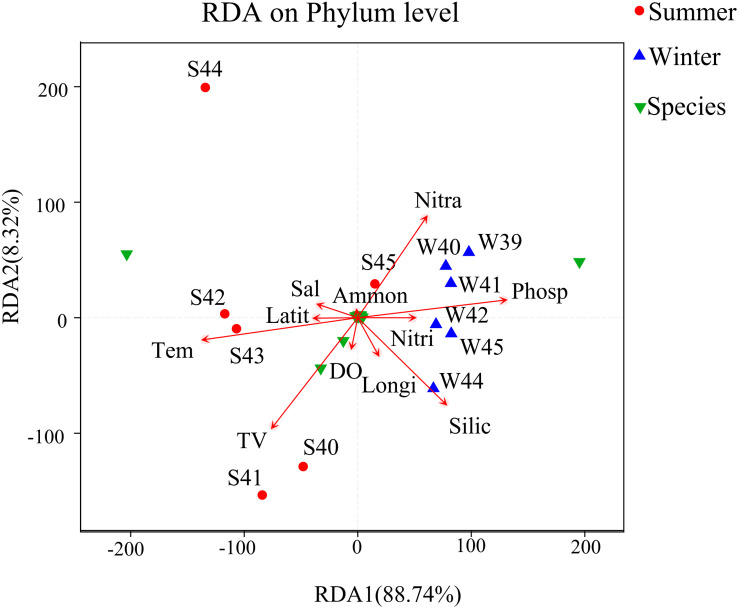
Redundancy analysis (RDA) between environmental factors, species, and samples at the phylum level was used to describe the influence of environmental factors on the bacterial community. Longi, longitude (°); Latit, latitude (°); TV, total virioplankton abundance (particles/mL); Tem, temperature (°C); Sal, salinity (‰); DO, dissolved oxygen (μmol/L); Phosp, PO_4_-P (μg/L); Nitra, NO_2_-N + NO_3_-N (μg/L); Ammon, NH_4_-N (μg/L); Nitri, NO_2_-N (μg/L); Silic, SiO_2_ (μg/L). Green triangles represent species of relative abundance in the top 30.

Correlation analysis among bacterial community structure, abundance, and environmental parameters were completed by RDA (Figure 7). From the result, *x*-axis and *y*-axis explained 88.74 and 8.32% of the variations in environmental factors and bacterial community in summer and winter. Temperature and phosphate may be the most important environmental factors that affected the bacterial community. Nitrate and phosphate were positively associated with the bacterial community. The RDA plot demonstrated that key environmental factors including temperature, silicate, phosphate, and nitrate shifted the bacterial community during two seasons.

**FIGURE 8:**
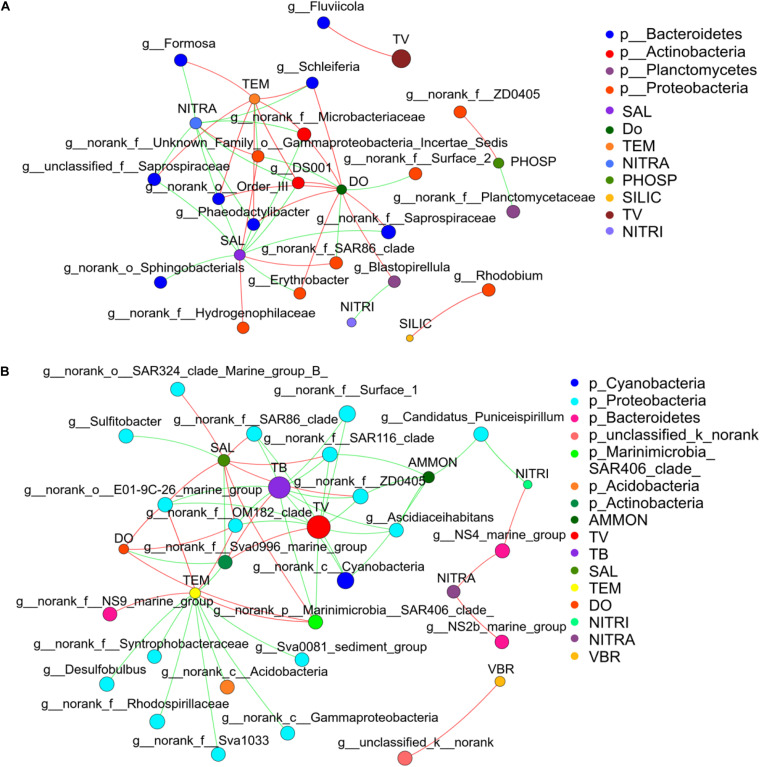
Microbial correlation network analysis in the middle of the Bohai Sea. **(A)** Network analysis showed strong correlations between bacterial community and environmental factors in summer. **(B)** Network analysis showed strong correlations between bacterial community and environmental factors in winter. Network analysis across the domain of bacteria was conducted based on the proportional OTU datasets. Each node represents the species at genus level and environmental factors, edge represents the correlations between environmental factors and bacterial species. Nodes size represent the relative abundance of each taxa. Nodes are colored by domains at the phylum level, and edges are colored by interaction types. Red line represents the positive correlation and green line represents the negative correlation. The thickness of the line indicates the size of the correlation coefficient, and the thicker the line, the higher the correlation between species (TEM, temperature; SAL, salinity; DO, dissolve oxygen; NITRA, nitrate; PHOSP, phosphate; SILIC, silicon; NITRI, nitrite; AMON, ammonium; TV, total virioplankton; TB, total bacterioplankton; VBR, virus to bacteria ratio).

Network analysis was used to compare how environmental parameters affected the bacterial community in winter and summer (Figures 8A,B) to further understand the relationship between bacterial community and environmental factors. The network diagram was pre-filtered, and only species with relative abundance in the top 50 were used to analyze the relationship with environmental factors. It was revealed that *Desulfobulbus* had a strong negative correlation with temperature in winter. *Ascidiaceihabitans* had a strong negative correlation with ammonium in winter. *Sulfitobacter* had a strong negative correlation with salinity in winter. In summer, *Erythrobacter* and *Phaeodactylibacter* had a negative correlation with salinity. *Formosa* had a positive correlation with temperature and showed negative relationship with nitrate. *Erythrobacter, Blastopirellula*, and *Schleiferia* had a positive correlation with DO. *Blastopirellula* had a negative correlation with nitrite. *Rhodobium* had a positive correlation with silicate. Totally, environmental factors such as temperature, salinity, and phosphate played paramount roles in controlling bacterial community (Supplementary Figure 7A).

### Prediction of Bacterial Community Structure and Function

**FIGURE 9:**
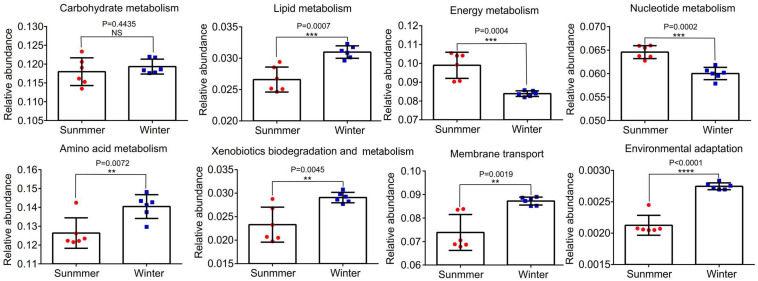
The relative abundance of the top eight functional groups of samples between summer and winter were analyzed. **P < 0.01, ***P < 0.001, ****P < 0.0001. Statistical *t*-test was used to detect whether there were significant differences in index values between two groups. Error bars show the standard deviation from triplicates samples.

Abundances of 16S rRNA gene OTUs were used to predict the function of bacterial community in the middle of the Bohai Sea. The relative abundances of the top eight functions were statistically analyzed. It was demonstrated that there were significant differences in the relative abundance of different samples between summer and winter (*P* < 0.05) (Figure 9). Functions related to carbohydrate metabolism, lipid metabolism, energy metabolism, nucleotide metabolism, amino acid metabolism, xenobiotics biodegradation and metabolism, membrane transport, and environmental adaptation were analyzed to determine the adaptation of bacterial community to the environmental variations. Abundance of functional bacterial communities associated with carbohydrate metabolism, lipid metabolism (*P* = 0.0007), amino acid metabolism (*P* = 0.0072), xenobiotics biodegradation and metabolism (*P* = 0.0045), membrane transport (*P* = 0.0019), and environmental adaptation (*P* = 0.0001) were significantly higher in winter than in summer. Relative abundance of energy metabolism (*P* = 0.0004) and nucleotide metabolism (*P* = 0.0002) were significantly lower in winter than in summer. Among all of the functions, the relative abundance of amino acid metabolism accounted for the largest proportion, followed by carbohydrate metabolism, energy metabolism, membrane transport, nucleotide metabolism, and lipid metabolism. Xenobiotics biodegradation and metabolism accounted for the smaller proportion in all functions.

## Discussion

In this study, the temporal and spatial dynamics of the bacterial community structure in the middle of the Bohai Sea under the influence of the estuarine output were investigated, and the relationship between environmental factors and the bacterial community structure and diversity was analyzed simultaneously, which may explain the bacterial distribution and diversity in the middle of the Bohai Sea from a new perspective.

Marine microorganisms play an important role in the process of the energy and material flux exchange. Therefore, it is important to figure out the relationships between the microbial community structure and environmental factors. Through the results, it was concluded that nutrient concentration presented a decreasing trend from the area near the estuary to the area far away from the estuary. Especially for the distribution of ammonium and nitrate, a decreasing trend was particularly pronounced (Figures 2B,C). The annual discharge flux of the Yellow River was the majority riverine output into the Bohai Sea which caused nutrient concentrations to change greatly (Zou et al., 2001). Especially during the summer, the abundant rainfall caused a large number of inland rivers to flood into the sea, and the increased discharge led to the extremely high eutrophication level of coastal waters (Wang et al., 2010). Compared with summer, the distribution of nutrients in winter also showed the highest nitrate concentration near the estuary, but the variation gradient from west to east was not obvious. In addition, the nitrate concentration in winter was significantly higher than that in summer (*P* < 0.01), while the ammonium concentration showed a decreasing trend compared with summer. The results demonstrated that the influence of estuarine output on the nutrient concentration varied greatly in two seasons (Nedwell et al., 2002). Inland rivers bring large amount of water with high concentrations of nitrate in the process of flowing into the coastal sea, causing a huge impact on the coastal ecosystem (Fabricius, 2005; Brodie et al., 2012).

The distribution characteristics of microplankton were closely related to environmental factors, seasonal changes, and biological interactions. Studies have indicated that both nutrient levels and organic matter compositions have a close correlation with bacterial community (Herlemann et al., 2017). It was showed that the high abundance of microplankton appeared near the estuary in winter, and the low abundance of virioplankton and bacterioplankton appeared near the estuary in summer (Figure 2E). From the correlation analysis, there were significant negative relationships between virioplankton and nutrient concentrations, while there were significant positive correlations with temperature (*P* < 0.01) (Figures 7, 8). Previous studies have also demonstrated that rising temperature, sufficient sunlight, and rich nutrient concentration in the coastal areas could cause a surge of large abundance of picoplankton which were thought to be the main reason to strengthen the competition between species (Amorim et al., 2016; Pulina et al., 2017). While in our study, the abundance of microplankton in the area far away from the estuary was much higher than the area near the estuary in summer, which verified the negative correlation between picoplankton and nutrient concentrations.

According to the analysis of the distribution of bacterial diversity among different samples, the bacterial diversity in the middle of the Bohai Sea showed a decreasing trend from west to east in both summer and winter. By the comparison between summer and winter, it showed that the richness index and diversity index in winter were significantly higher than that in summer (Figure 3). It was common that the Chao1 index and Shannon index were significantly higher in summer than in winter because of high temperature, sufficient light, and nutrition (Mohapatra et al., 2020). It has also been reported that the diversity of planktonic bacteria in the south Adriatic Sea area varied greatly with the change of seasons, and the diversity index in summer was significantly higher than other seasons (Korlevic et al., 2016). However, in the eutrophic area such as the English Channel region, it was found that the high richness and diversity index appeared in winter by comparing with summer (Gilbert et al., 2012), which was consistent with our results. High bacterial diversity was often associated with nutrient, temperature cycle, phytoplankton, and zooplankton (Jürgens et al., 1999). This suggests that, as productivity and nutrients increased, these bacteria also increased in abundance, that is, these taxa appear to perform best in a productive system (Gilbert et al., 2012). Our study showed an increase in diversity during winter. One of the reasons for this diversity distribution pattern may be the effect of the estuary output on the coastal waters. Estuarine output was not only a constant flux, but also varied with environmental changes, sometimes in a short period or with seasonal changes and different spatial scales, leading to different trends in microbial diversity distribution (Barz et al., 2010; Ghiglione and Murray, 2012). The conditions with winter mixing were rarely a productive period when certain species can quickly take up nutrients may explain the sharp increase in richness (Hernando-Morales et al., 2018).

Furthermore, there were significant differences in the composition of bacterial community between summer and winter according to the results of microbial community diversity (*P* < 0.05). At the phylum level, the dominant species in summer was Cyanobacteria, and the dominant species in winter was Proteobacteria. At the class level, the dominant species in summer was Cyanobacteria, and the dominant species in winter was Gammaproteobacteria. At the genus level, the dominant species in summer was *Synechococcus*, while the dominant species in winter was *Pseudoalteromonas*. It was reported that Proteobacteria was ubiquitous in marine environment. Our results were consistent with previous studies in coastal marine water (Xie et al., 2014; Zhang et al., 2014). It has been investigated that the abundance of Alphaproteobacteria and Gammaproteobacteria in seawater in winter increased obviously, which might be an important reason for the higher microbial diversity index in winter than in summer. The geolocation in this study is similar to the Baltic, which is one of the largest environments with pronounced salinity gradient (Rojas-Jimenez et al., 2019). It was suggested that rare taxa could contribute greatly to microbial community dynamics when environmental conditions were suitable (Shade et al., 2014). Some dormant bacteria could be triggered to regrow when dispersed into new salinity conditions (Shen et al., 2018b). However, it was not clear whether or not some bacteria were dispersed into the Bohai Sea from the river outlet, in terms of seed bank, or functionally equivalent taxa. In view of the distribution character of microorganisms in the middle of the Bohai Sea, we compared taxa distribution near the Yellow River outlet and the middle of the Bohai Sea. Many species such as Rhodobacteriaceae, Actinomarina, Saprospiraceae, Flavobacteria, and *Planctomyces* presented a higher relative abundance near the estuary than the middle of the Bohai Sea. It was demonstrated that Flavobacteria was widely distributed and isolated from the freshwater soil (Mccammon and Bowman, 2000). In this study, several families within the Flavobacteria were present in high relative abundance near the estuary, which verified our hypothesis that some bacteria inhabiting Yellow River outlet may be flowed into the saline Bohai Sea. Definite evidence needs to be verified in the following work.

Alphaproteobacteria has been considered to be strongly sensitive to marine water environmental variation, especially sensitive to salinity (Grommen et al., 2005). It has been proved that patterns of bacterial community both in winter and summer were strongly impacted by salinity in the Baltic Sea (Herlemann et al., 2016). In our results, high correlations were also found between Alphaproteobacteria and salinity, suggesting that salinity played a pivotal role in impacting bacterial taxa abundance (Herlemann et al., 2011; Shen et al., 2018a). Gammaproteobacteria was one taxon that played an important role in pollution degradation in the marine environment (Wu et al., 2014; Zhang et al., 2014). Many studies had showed that Gammaproteobacteria was more suitable to cold environments and vulnerably affected by salinity (Rocker et al., 2012; Korlevic et al., 2016; Franco et al., 2017). While in our study, results indicated that Gammaproteobacteria may not be suitable for living in high-salt environments but it was easy to survive in high nitrate and phosphate concentration environment (Hoeft et al., 2007; de Leon-Lorenzana et al., 2017; Moisander et al., 2018). Sphingobacteria has been reported to have a significant positive correlation with concentrations of phosphorus and nitrogen which suggested that these bacterial group may participate in the organic matter degradation (Sinkko et al., 2013). In our study, Sphingobacteria showed a significant negative correlation with salinity and phosphorus, and showed a significant positive correlation with temperature and concentration of ammonium (Supplementary Figure 7B). These results were partially consistent with previous studies. However, high abundance of Cyanobacteria was found in summer which was consistent with what was observed in earlier study (Barz et al., 2010). The high abundance of Cyanobacteria was found to be highly impacted by temperature, salinity, and phosphate, which likely promoted the growth of Cyanobacteria in the coastal sea (Zhou et al., 2016).

Temperature (*P* < 0.01), phosphate (*P* < 0.01), nitrate (*P* < 0.05), and silicate (*P* < 0.05) concentrations were found to be strongly correlated with bacterial species distribution according to the RDA analysis and spearman correlation analysis. There was a significant negative correlation between temperature and most of the bacterial species distribution (*P* < 0.01). However, there was a significant positive correlation with Cyanobacteria and Bacteroidetes abundance, which was consistent with previous studies in the NW Mediterranean Sea (Díez-Vives et al., 2014). Focusing on the taxonomic data obtained from this study, *Fluviicola* was considered to be mesophilic type strain, which was identified from the genus level (Supplementary Figure 2D; O’Sullivan et al., 2004). In addition, high relative abundance of *Synechococcus* was found at the genus level of bar plot (Supplementary Figure 2D). It had been proved that temperature was an important factor in controlling photosynthesis rate, and the occurrence of *Synechococcus* had been linked to temperature (Zwirglmaier et al., 2008). Thus, the presence of these key phylotypes may partially explain the positive relationship with temperature. However, phosphate showed an obvious positive correlation with the distribution of species (*P* < 0.01). Proteobacteria had a significant positive correlation with salinity and phosphate concentration. Phosphate concentration was significantly higher in winter than in summer, which might be an important reason of the dominant position of Proteobacteria in winter.

Tax4Fun function analysis predicted that carbohydrate metabolism, lipid metabolism, amino acid metabolism, xenobiotics biodegradation and metabolism, membrane transport, and environmental adaptation presented a much higher trend in winter (Figure 9). This was consistent with previous studies on the microbial community in river outlets of the subtropical Pearl River Estuary (Mai et al., 2018). Characteristics of functional genes distribution suggested that the function of bacterial communities in winter may be highly correlated with environmental stresses such as low temperature (Dai et al., 2016). The relative abundance of energy metabolism was much lower in winter than in summer may be also due to the low temperature that reduced the enzyme activity (Santoro et al., 2008). The activity of DNA polymerases and nucleotide metabolism were easily inhibited by the low temperature which may help explain this phenomenon (Fortunato and Crump, 2015). Finally, stresses caused by variations in the temporal and spatial environment may exacerbate interspecific and intraspecific competition within bacterial communities, resulting in the occurrence of a large abundance of functional genes associated with environmental information (Aimée et al., 2016). These preliminary results should be confirmed by whole metagenome profiling in the following work via sufficient geochemical analyses to better link taxonomic and biogeochemical functions in the middle of the Bohai Sea.

## Conclusion

Maintaining the stability of the diversity of marine microbial community is of great significance to the balanced development of marine ecosystem. In this study, spatial and temporal variation of microbial community in the subtropical coastal Bohai Sea was investigated to study the impact of estuarine output. Among all of the environmental parameters, temperature, salinity, nitrate concentration, and phosphate concentration had important effects on the structure of microbial community. In particular, these environmental variations caused by estuarine output had significant effects on Proteobacteria, Marinimicrobia_SAR406_clade, Nitrospinae, Acidobacteria, Nitrospirae, Deferribacteres, lgnavibacteriae, Latescibacteria, Gemmatimonadetes, and Chloroflexi. In addition, the diversity of microbial community structure in winter was much more complex than in summer which might be related to different emission flux transported by the estuarine output all year round. And microbial community structure varied with the environment change presented its unique complexity and diversity. Studies on the coastal microbial community structure and diversity would enhance our ability to elucidate their relationships with environmental variation. This study will provide a theoretical basis for further work on the effects of estuarine output and seasonal variation on microbial community dynamics and their functional outcomes.

## Data Availability Statement

The datasets presented in this study can be found in online repositories. The names of the repository/repositories and accession number(s) can be found in the article/Supplementary Material.

## Author Contributions

CW wrote and revised the manuscript. HZ and PL collected samples and revised the manuscript. YW collected samples. YS and ZS collected samples and analyzed samples. XH supervised the whole experiment and revised the manuscript. All authors contributed to the article and approved the submitted version.

## Conflict of Interest

The authors declare that the research was conducted in the absence of any commercial or financial relationships that could be construed as a potential conflict of interest.

## Footnotes

1 http://qiime.org/scripts/split_libraries_fastq.html

2 http://www.mothur.org/wiki/Schloss_SOP#Alpha_diversity
