# Supplementary material for: Divergent Patterns of Bacterial Community Structure and Function in Response to Estuarine Output in the Middle of the Bohai Sea
Source: Front Microbiol. 2021 Mar 8;12:630741. doi: 10.3389/fmicb.2021.630741 (PMC7982528; doi:10.3389/fmicb.2021.630741)
Supplement: Supplementary file 1 [file Data_Sheet_1.docx]

Supplementary Material

# Supplementary Data

**Figure S1.** Seasonal variation of alpha diversity in the study area. Differences between winter and summer were showed by α diversity index (Chao1 index, Shannon index, Simpson index and Ace index). Wilcoxon rank-sum test was used to examine differences between groups. ***P*<0.01, **P*<0.05.

**Figure S2** A: Bacterial community composition at the class level. B: Bacterial community composition at the order level. C: Bacterial community composition at the family level. D: Bacterial community composition at the genus level.

**Figure S3** Hierarchical clustering tree on OTU level.

**Figure S4** PcoA analysis on OTU level.

**Figure S5** Differences between species in summer and winter at the class level based on Wilcoxon rank-sum test.

**Figure S6** Linear discriminate analysis (LDA) was used to compare differences between summer and winter (LDA score<4).

**Figure S7** Spearman correlation heatmap was used to analyze the relationships between species and environmental factors at the phylum level (A) the order level (B). (Tem: temperature; Ammon: ammonium; TV: total virioplankton abundance; TB: total bacterioplankton abundance; Sal: salinity; Phosp: phosphate; Nitra: nitrate; VBR: virus to bacteria ratio; Nitri: nitrite; Silic: silicon) ** *P*<0.01, * *P*<0.05.


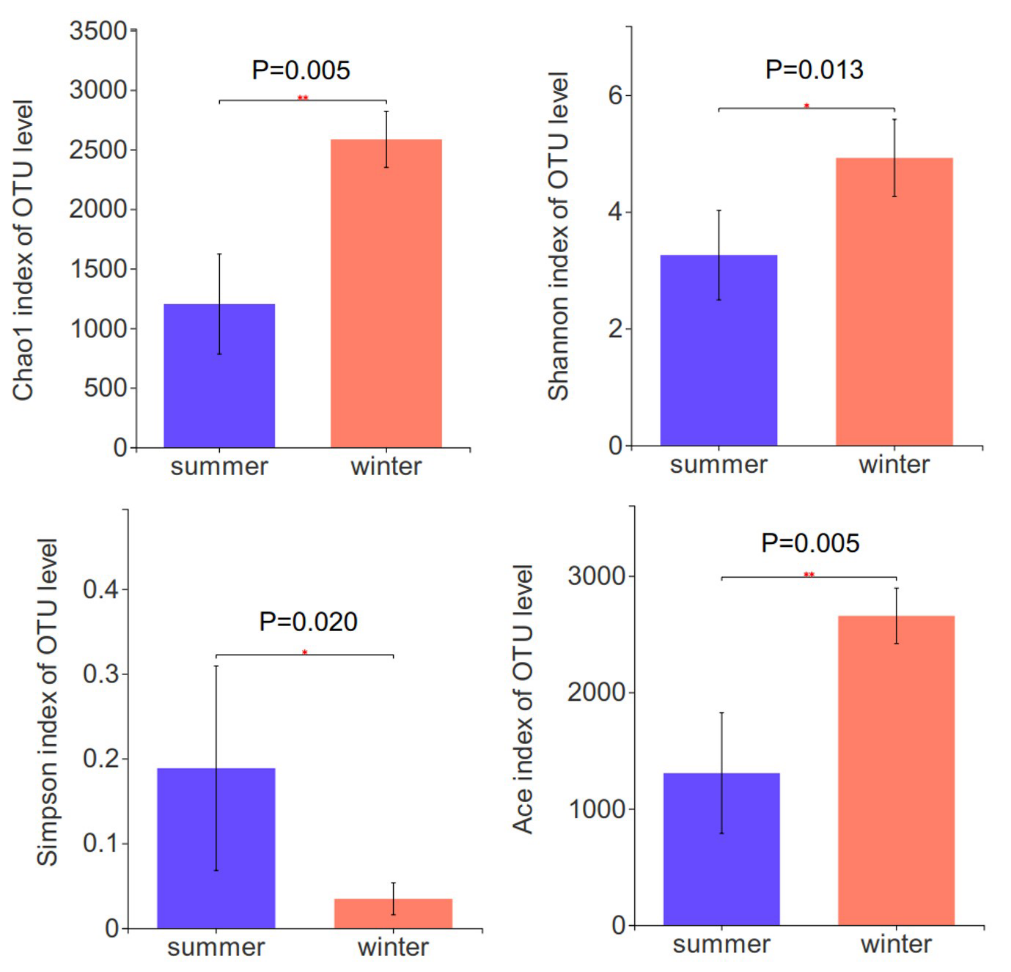


Figure S1


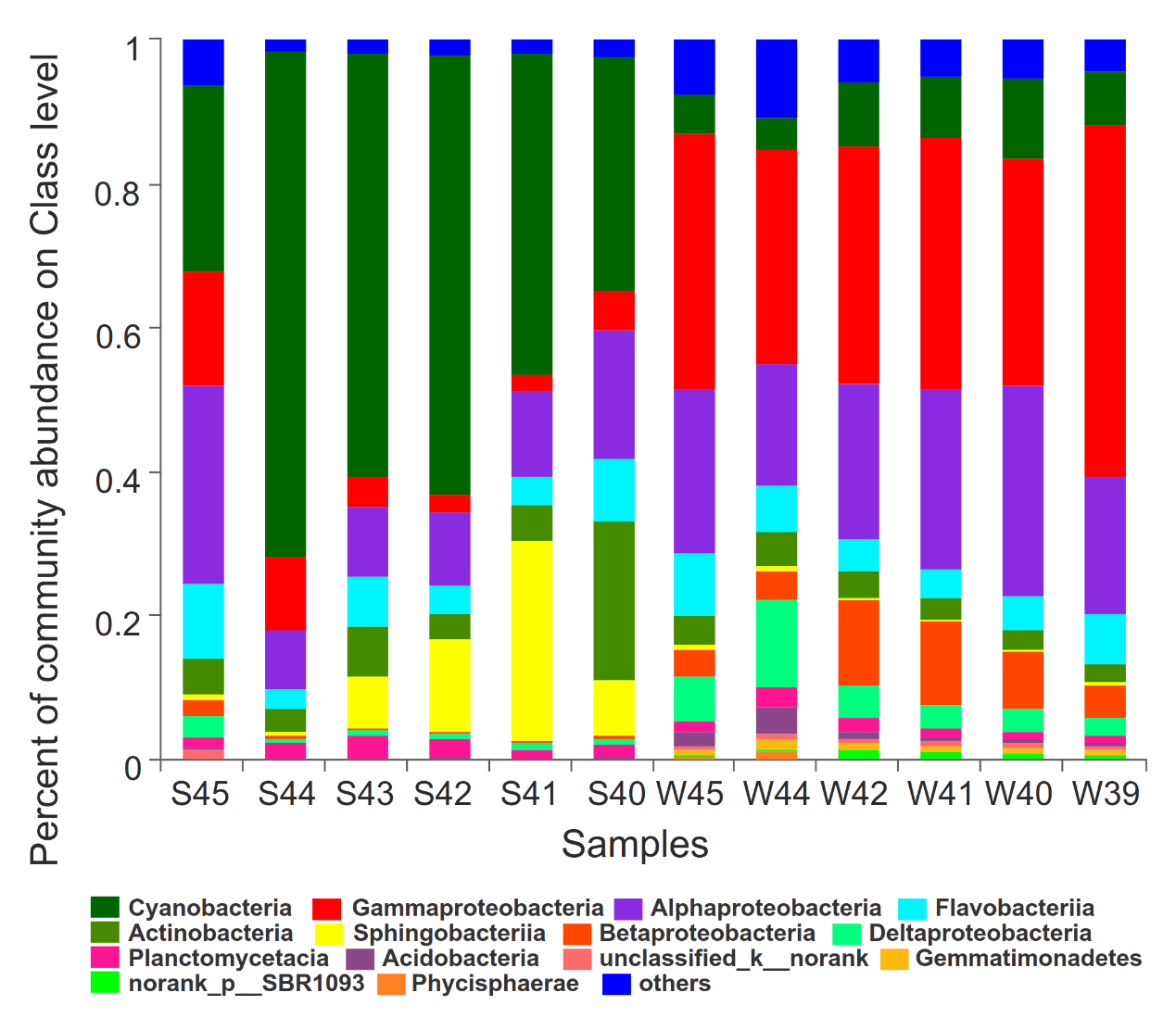


Figure S2A
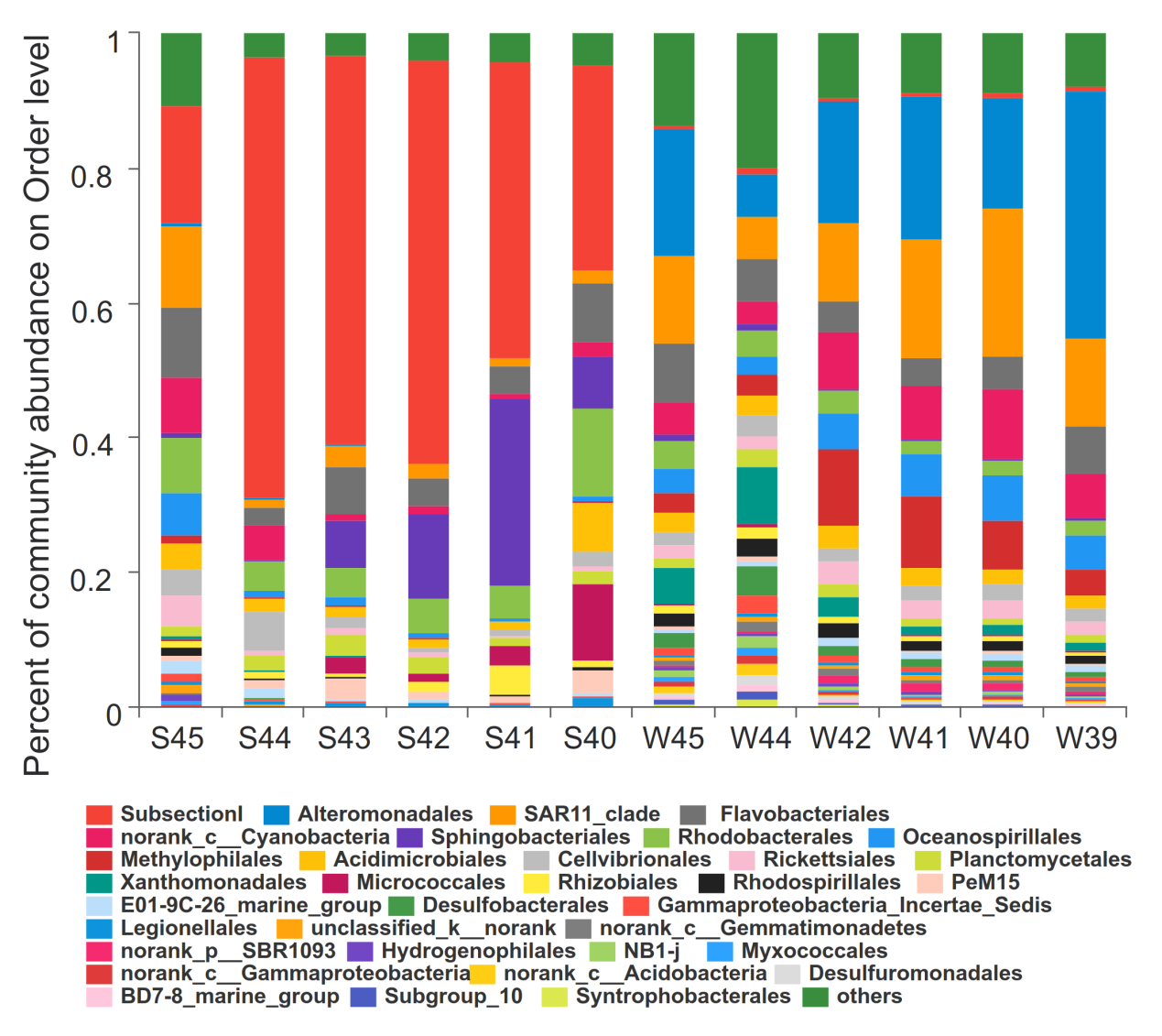
Figure S2B


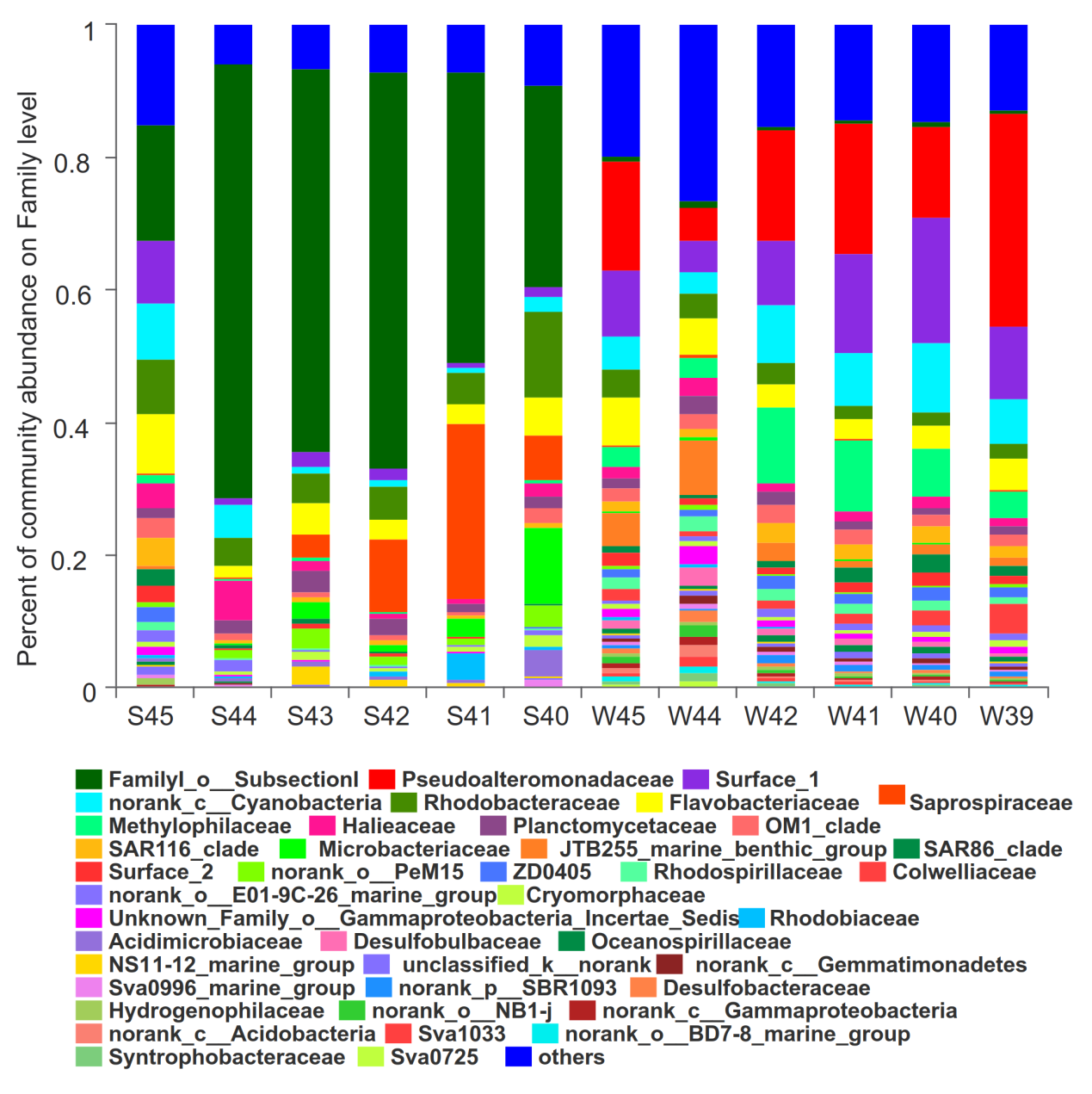
Figure S2C


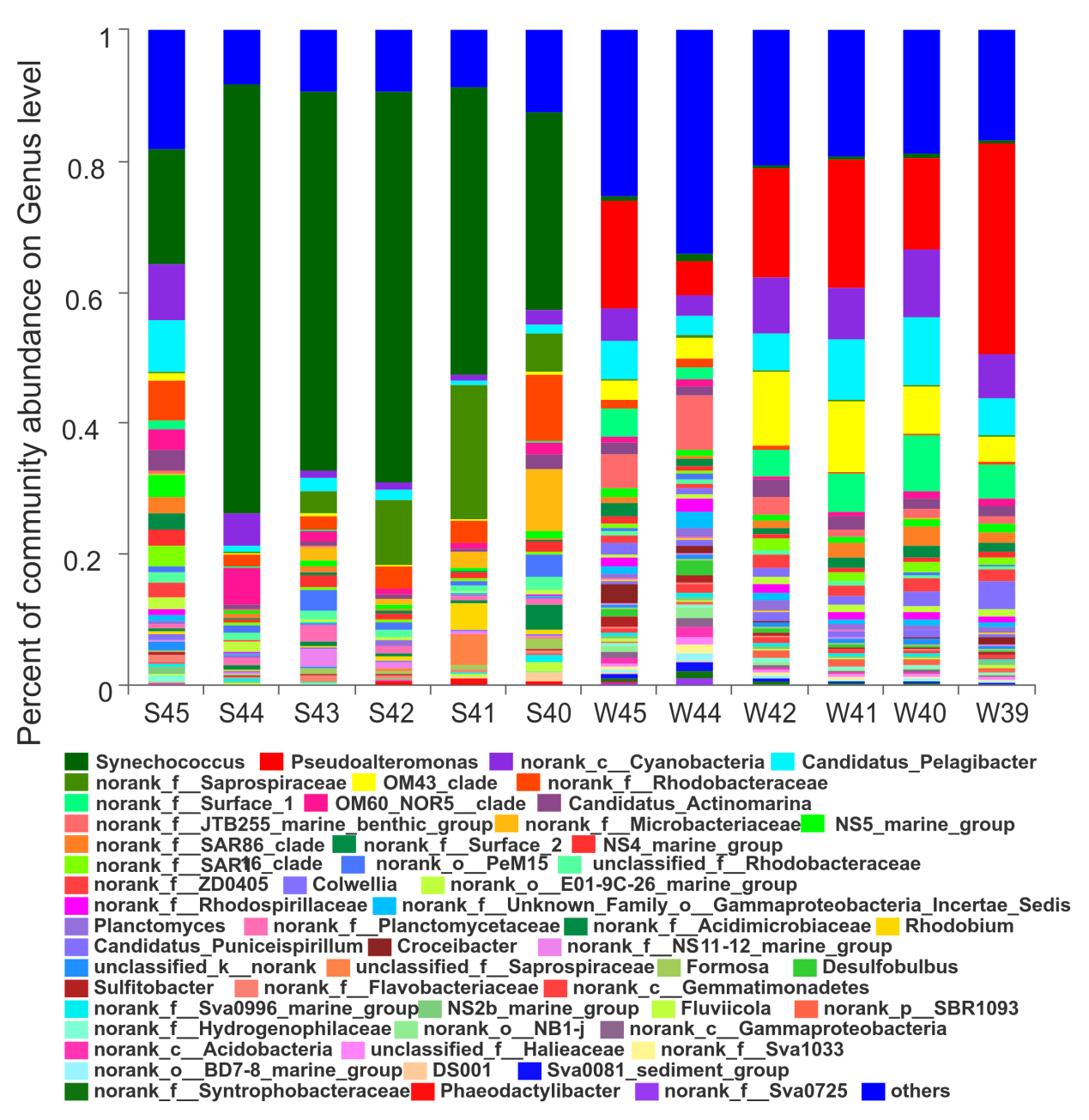
Figure S2D


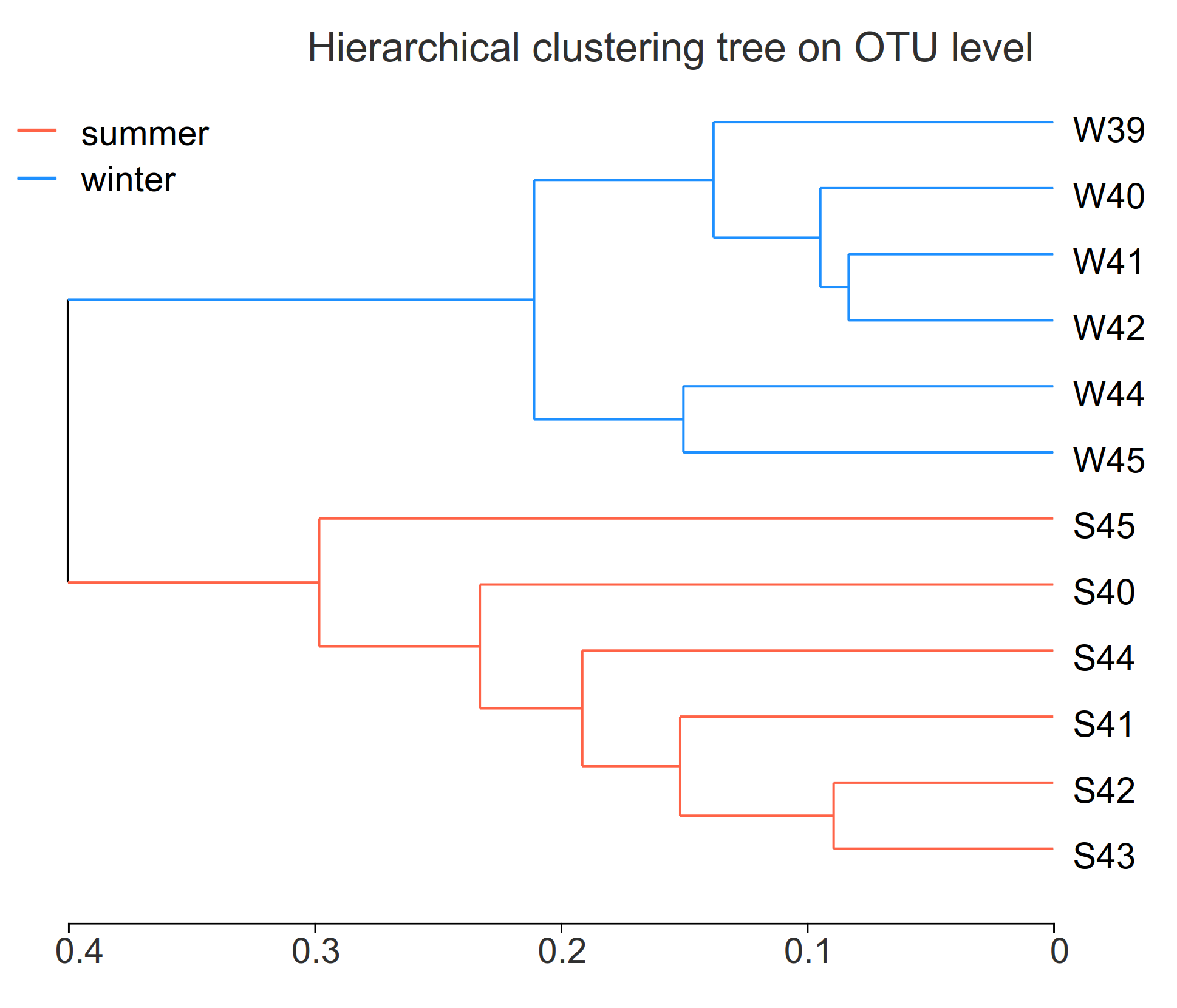
Figure S3


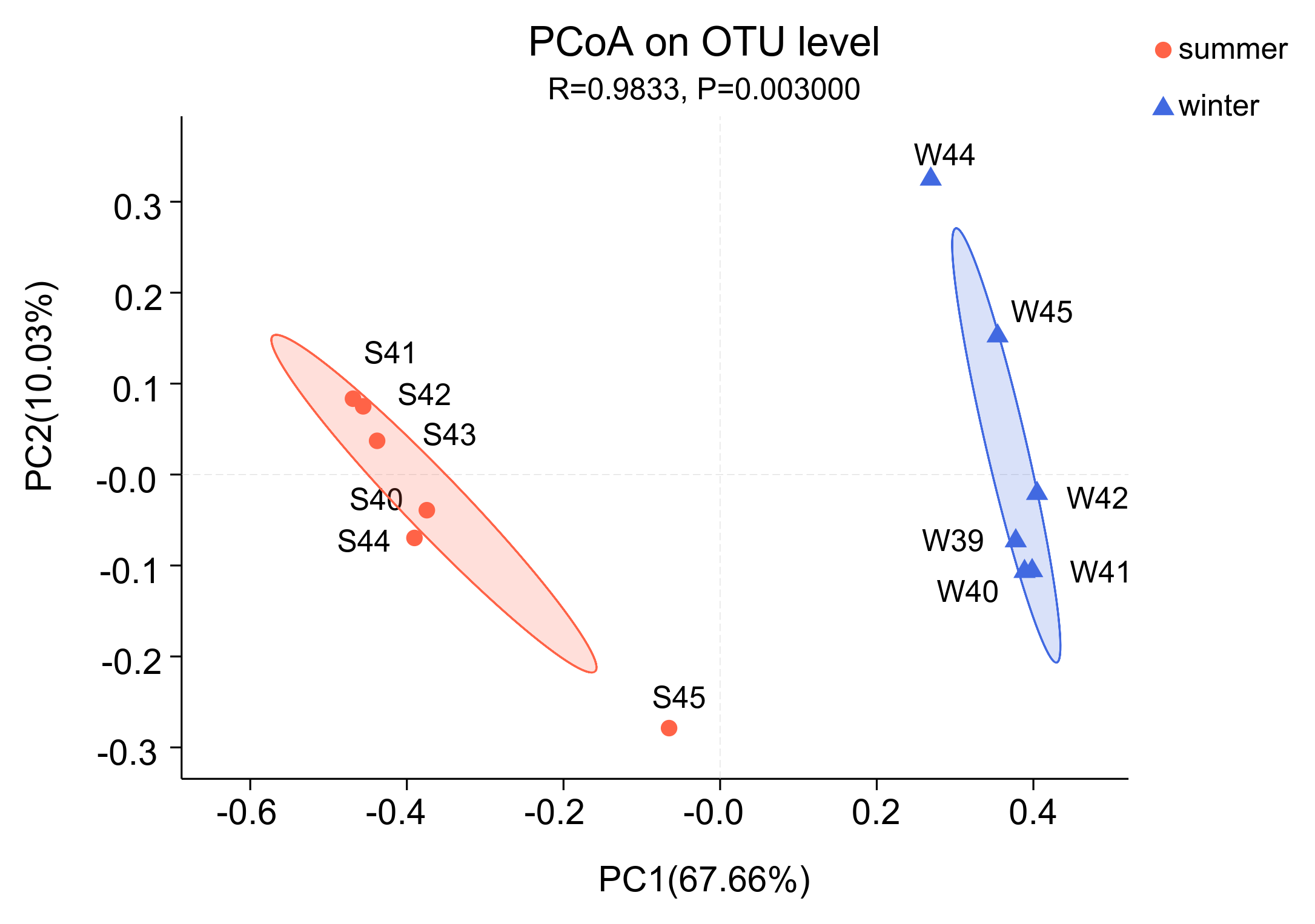
Figure S4


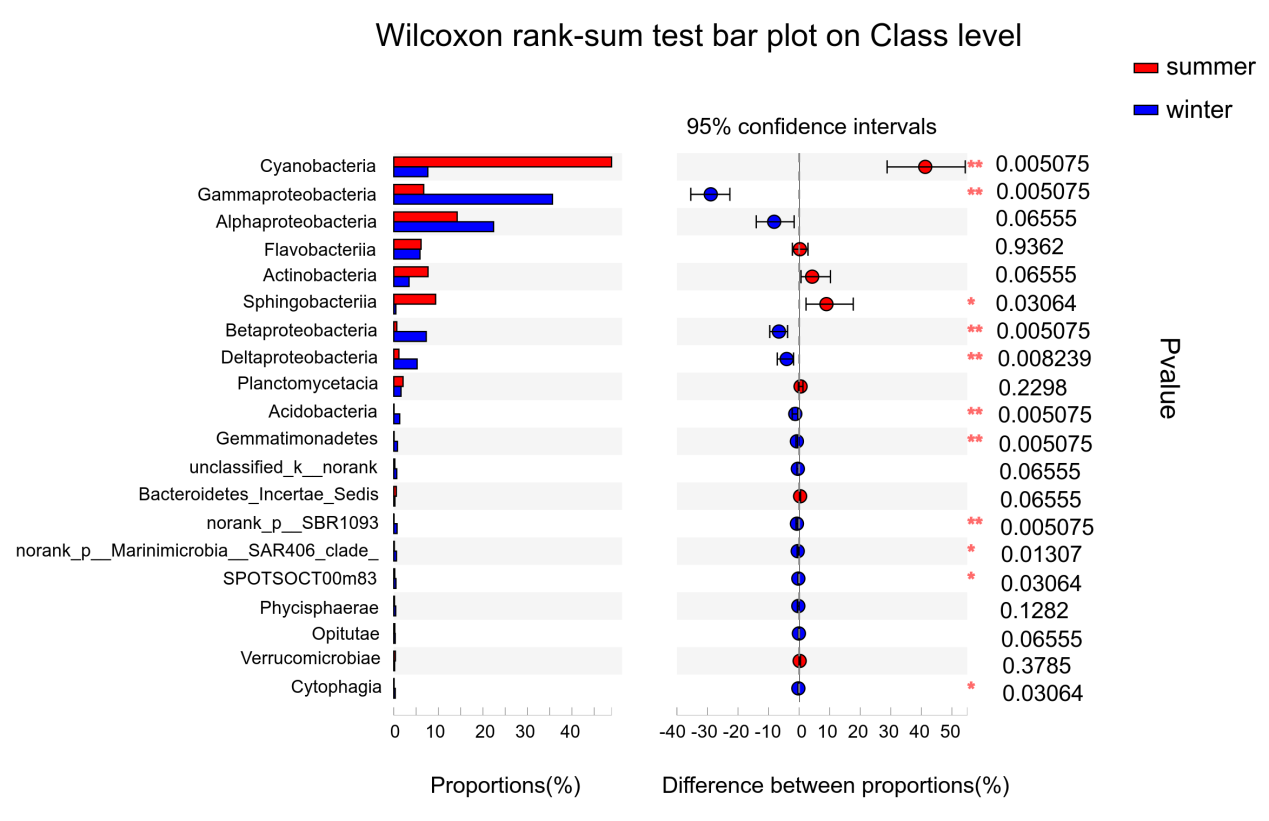
Figure S5


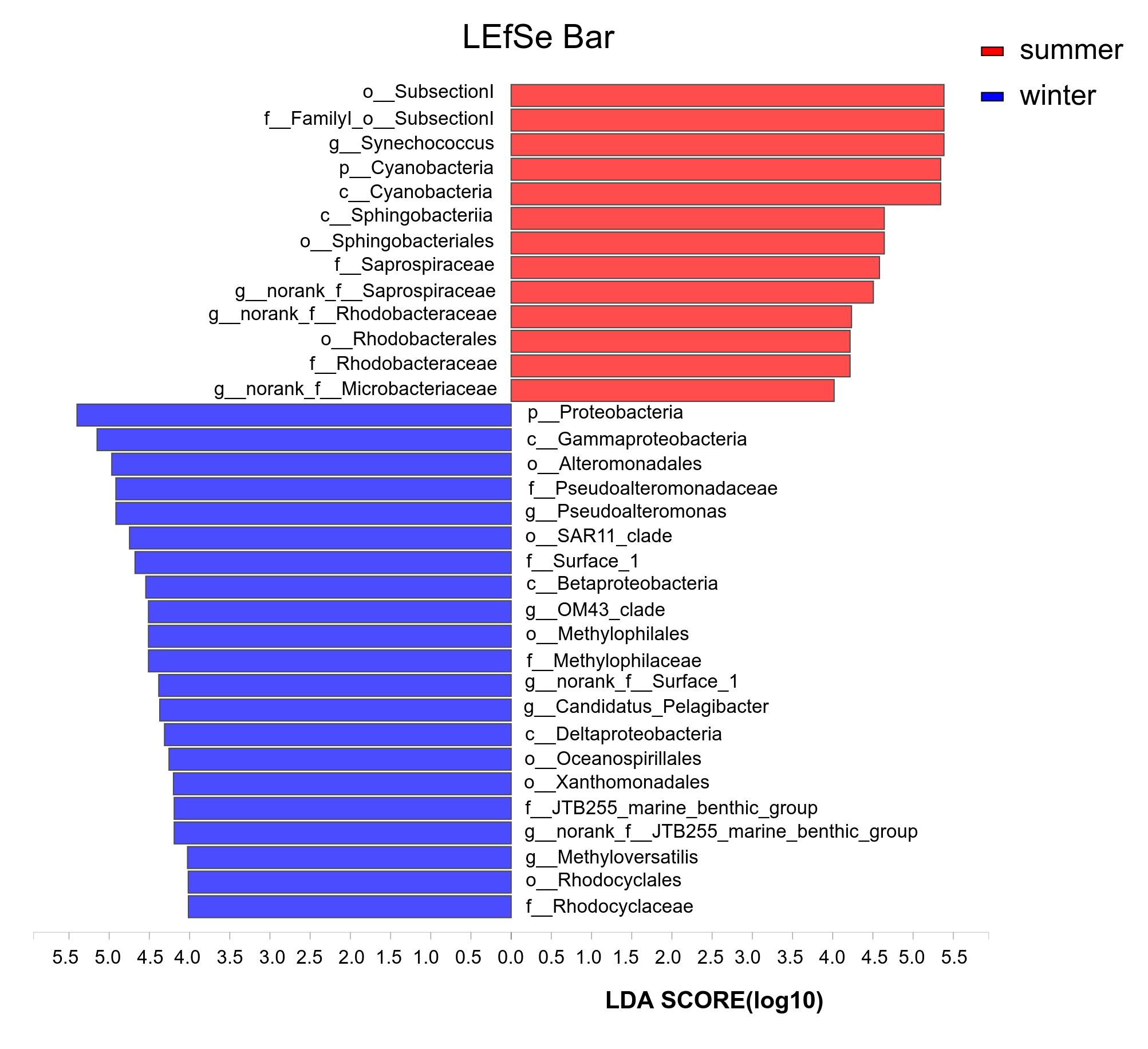
Figure S6


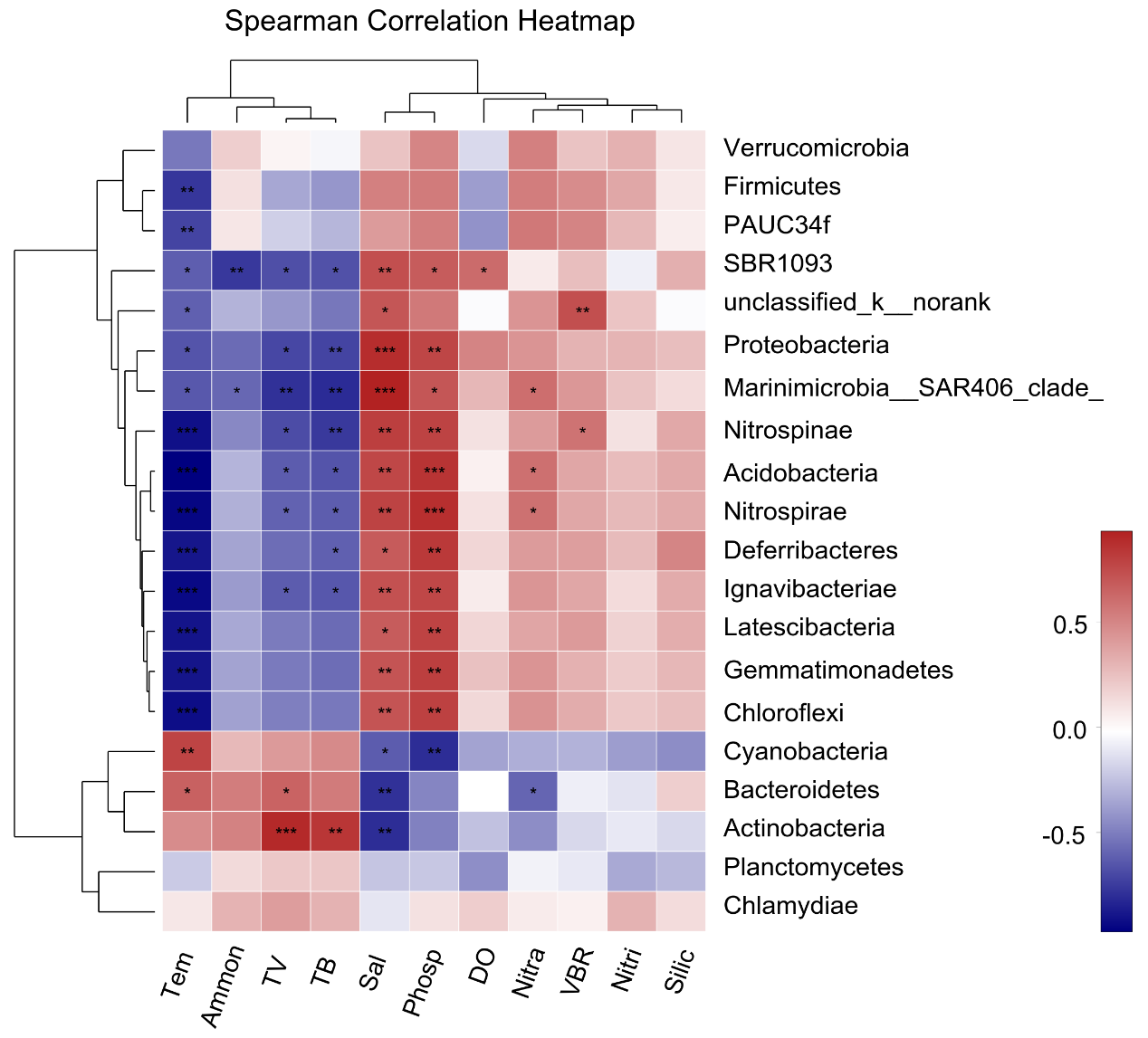


Figure S7A


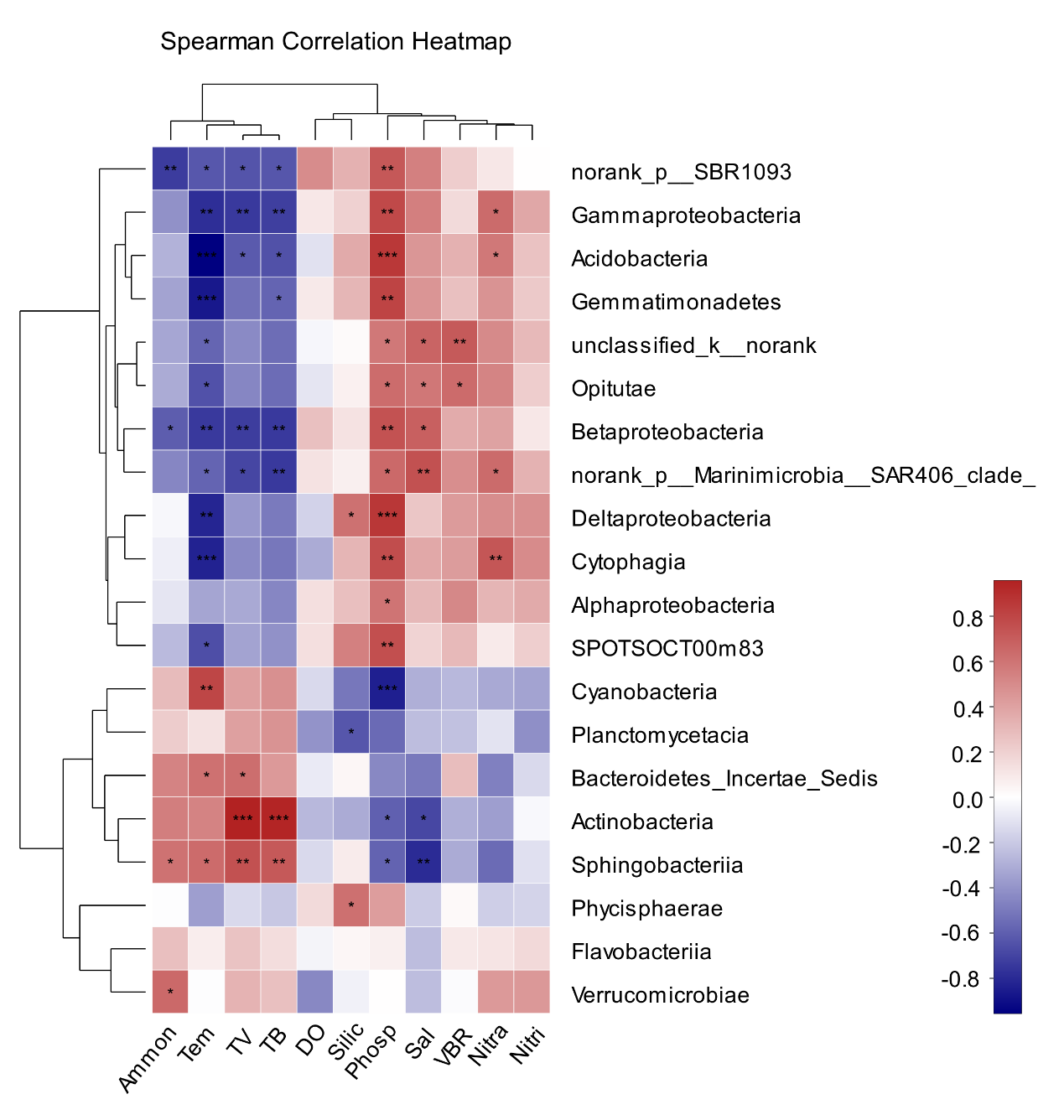


Figure S7B

# Supplementary table

**Table S1** Environmental parameters of all stations

| Station | Longi | | Latit | Dep | Tem | Sal | DO | Phosp | Nitra | Ammon | Nitri | Silic | TV(×10^6^) | TB(×10^5^) | VBR |
| --- | --- | --- | --- | --- | --- | --- | --- | --- | --- | --- | --- | --- | --- | --- | --- |
| S45 | 119.00 | 38.32 | | 3 | 24.28 | 30.99 | 5.27 | 10.32 | 91.60 | 114.92 | 6.85 | 0.35 | 1.87 | 8.71 | 21.53 |
| S44 | 119.20 | 38.32 | | 3 | 24.06 | 31.03 | 6.03 | 8.95 | 103.50 | 14.18 | 1.85 | 2.00 | 5.95 | 10.78 | 5.52 |
| S43 | 119.44 | 38.32 | | 3 | 25.25 | 30.90 | 6.76 | 5.47 | 53.28 | 9.97 | 0.85 | 0.19 | 13.68 | 20.03 | 6.83 |
| S42 | 119.78 | 38.35 | | 3 | 25.45 | 30.87 | 7.02 | 7.18 | 50.48 | 25.64 | 0.57 | 0.39 | 5.50 | 6.09 | 9.02 |
| S41 | 120.18 | 38.33 | | 3 | 25.74 | 30.21 | 7.72 | 9.26 | 39.12 | 16.88 | 2.29 | 0.56 | 24.38 | 38.03 | 6.41 |
| S40 | 120.46 | 38.34 | | 3 | 25.84 | 30.79 | 10.43 | 7.64 | 12.04 | 11.53 | 0.85 | 0.34 | 27.03 | 43.32 | 6.24 |
| W45 | 118.60 | 38.19 | | 3 | 0.72 | 31.21 | 7.10 | 22.74 | 147.58 | 56.99 | 18.51 | 0.55 | 3.38 | 6.12 | 5.52 |
| W44 | 119.20 | 38.32 | | 3 | 0.60 | 31.51 | 7.23 | 17.82 | 65.96 | 11.31 | 1.81 | 0.45 | 6.45 | 6.78 | 9.51 |
| W42 | 119.78 | 38.34 | | 3 | 1.43 | 32.16 | 8.02 | 17.91 | 82.94 | 0.56 | 1.29 | 0.45 | 2.41 | 3.09 | 7.78 |
| W41 | 120.19 | 38.33 | | 3 | 1.83 | 32.16 | 7.59 | 13.47 | 56.08 | 6.26 | 1.42 | 0.33 | 2.88 | 3.14 | 9.17 |
| W40 | 120.45 | 38.34 | | 3 | 2.20 | 32.28 | 8.54 | 20.81 | 83.13 | 1.58 | 1.41 | 0.48 | 1.22 | 1.26 | 9.70 |
| W39 | 120.71 | 38.34 | | 3 | 4.46 | 32.25 | 10.36 | 16.61 | 87.84 | 2.63 | 2.16 | 0.42 | 1.98 | 2.63 | 7.53 |

Abbr: Longi: Longitude (°); Latit: Latitude (°); Dep: Depth (m); Tem: Temperature (°C); Sal: Salinity (‰); DO: Dissolved oxygen (μmol/L); Phosp: PO_4_-P (μg/L); Nitra: NO_2_-N+NO_3_-N (μg/L); Ammon: NH_4_-N (μg/L); Nitri: NO_2_-N (μg/L); Silic: SiO_2_ (μg/L); TV: Total virus-like particle (particles/mL); TB: Total heterotrophic bacteria (cells/mL); VBR: Virus: bacteria Ratio. The assay was carried out as previously reported [1].

**Reference**

1. Liu, J.W., Zhu, S.Q., Liu, X.Y., Yao, P., Ge, T.T., and Zhang, X.H. (2020). Spatiotemporal dynamics of the archaeal community in coastal sediments: assembly process and co-occurrence relationship. ISME J. 14(6): 1-16. doi: 10.1038/s41396-020-0621-7
